# Effects of intermittent theta-burst stimulation on cognition and glymphatic system activity in mild cognitive impairment and very mild Alzheimer’s disease: a randomized controlled trial

**DOI:** 10.1186/s12984-025-01738-1

**Published:** 2025-09-26

**Authors:** Cheng-Chang Yang, Ko-Yen Huang, Jung-Lung Hsu, Chaur-Jong Hu, Yueh-Hsun Lu, Yi-Chun Kuan

**Affiliations:** 1 Department of Neurology, Shuang-Ho Hospital, Taipei Medical University, No. 291, Zhongzheng Rd., Zhonghe District, New Taipei City, 235041 Taiwan; 2 Department of Clinical Psychology, College of Medicine, Fu Jen Catholic University, New Taipei City, 242062 Taiwan; 3 Department of Physical Medicine and Rehabilitation, Shuang-Ho Hospital, Taipei Medical University, New Taipei City, 235041 Taiwan; 4 Department of Neurology, New Taipei Municipal Tu Cheng Hospital, New Taipei City, Taiwan; 5 Department of Neurology, Neuroscience Research Center, and College of Medicine, Chang Gung Memorial Hospital Linkou Medical Center, Chang-Gung University, Taoyuan, Taiwan; 6 Department of Neurology, School of Medicine, College of Medicine, Taipei Medical University, No. 250, Wuxing St., Xinyi Dist, Taipei, 110301 Taiwan; 7 Taipei Neuroscience Institute, Taipei Medical University, Taipei, 110301 Taiwan; 8 Dementia Center, Shuang-Ho Hospital, Taipei Medical University, New Taipei City, 235041 Taiwan; 9 Department of Radiology, Shuang-Ho Hospital, Taipei Medical University, New Taipei City, 235041 Taiwan; 10 Department of Radiology, School of Medicine, College of Medicine, Taipei Medical University, Taipei, 11031 Taiwan; 11 Department of Biomedical Engineering, National Taiwan University, Taipei, 100233 Taiwan

**Keywords:** Intermittent theta burst stimulation, Transcranial magnetic stimulation, Mild cognitive impairment, Alzheimer’s disease, Glymphatic system, Diffusion tensor image analysis along the perivascular space (DTI-ALPS)

## Abstract

**Background:**

Repetitive transcranial magnetic stimulation (rTMS) has demonstrated efficacy in alleviating cognitive symptoms in Alzheimer’s disease (AD) and mild cognitive impairment (MCI). Although animal studies suggest rTMS may enhance glymphatic system efficiency and reduce amyloid deposits, its impact on human glymphatic activity remains uncertain.

**Methods:**

This double-blind, randomized, sham-controlled trial investigated the effects of intermittent theta-burst stimulation (iTBS), a novel rTMS technique, on cognitive function and glymphatic system activity using diffusion tensor image analysis along the perivascular space (DTI-ALPS) in 52 participants with amnestic MCI or very mild AD. Participants underwent 10 sessions of iTBS targeting the left dorsolateral prefrontal cortex over two weeks. Cognitive and glymphatic assessments were conducted at baseline, week 2, and week 6. Of the 52 participants, 28 received active iTBS, while 24 received sham stimulation. After the first two weeks, the sham group transitioned to active iTBS.

**Results:**

Significant cognitive improvements were observed at week 6 in the iTBS group, indicating delayed cognitive enhancement, though no immediate changes in cognition or glymphatic system activity (measured by the ALPS index) were observed. No adverse events were reported.

**Conclusions:**

These findings suggest that iTBS can produce delayed cognitive enhancement in individuals with amnestic MCI and very mild AD, while the impact on the glymphatic system remains uncertain and requires further investigation.

**Trial registration:**

Clinicaltrials.gov (NCT04555941).

## Background

Alzheimer’s disease (AD), the leading cause of dementia, progressively impairs cognitive and behavioral functions, severely affecting daily activities [1]. Amnestic mild cognitive impairment (MCI) is considered a precursor to AD, affecting 20–30% of the elderly, with an annual conversion rate to AD of approximately 15% [2–4]. The global dementia population is expected to rise from 55 million to 139 million by 2050 [5]. While current pharmacological treatments offer symptomatic relief, they may be limited by side effects and their challenges in slowing disease progression, particularly given the accelerated cognitive decline observed in people with dementia after the COVID-19 pandemic [1, 6, 7].

Repetitive transcranial magnetic stimulation (rTMS) has emerged as a promising therapy for alleviating symptoms in individuals with AD and MCI [8]. High-frequency rTMS (HF-rTMS) has been demonstrated to improve cognition, with benefits lasting for at least one month [8, 9]. Intermittent theta-burst stimulation (iTBS), a newer form of HF-rTMS, offers advantages such as shorter stimulation duration, lower stimulation intensity, prolonged therapeutic effects, and reduced side effects [10, 11]. iTBS induces long-term changes in brain activity by enhancing neuroplasticity and triggering long-term potentiation (LTP)-like effects on cortical synapses [12–14]. Since LTP-like neuroplasticity is impaired in AD [15, 16], iTBS could represent a new therapeutic approach for treating cognitive impairment in AD [17].

One key advantage of HF-rTMS is its delayed cognitive effects, which may take several weeks to fully manifest. Although iTBS has shown promise for cognitive enhancement, studies specifically examining its delayed effects in MCI and AD are sparse [18], and there remains considerable variability in the application of rTMS protocols [19, 20]. A recent systematic review and network meta-analysis revealed that HF-rTMS yields greater delayed cognitive enhancement in individuals with AD and MCI compared to immediate effects [8]. This suggests that the neuromodulatory impact of rTMS may be gradual, with some participants experiencing therapeutic benefits weeks after treatment as synaptic and neuroplastic changes accumulate.

The glymphatic system, a recently identified waste clearance pathway, facilitates cerebrospinal fluid flow along the perivascular space through the brain and into the interstitial space [21–23]. Disruption of this system is believed to contributes to the accumulation of neurotoxic waste products, such as amyloid and tau proteins, both of which are key factors in AD pathology [24, 25]. However, the relationship between glymphatic activity and cognitive outcomes from rTMS remains unclear. Although some animal studies suggest that rTMS could enhance glymphatic efficiency, the relevance of this in humans is still under investigation [26].

Traditionally, rTMS’s effects on cognition have been captured through tools like the Mini-Mental State Examination (MMSE) [27] and Montreal Cognitive Assessment (MoCA) [28]. While diffusion tensor image analysis along the perivascular space (DTI-ALPS) has been explored as a non-invasive, quantitative measure of glymphatic activity [29, 30], its role in understanding rTMS’s cognitive augmentation effects remains unclear, despite some positive findings in AD animal models [31].

Despite growing interest in rTMS’s ability to modulate cognition over time, the connection between delayed cognitive benefits and underlying mechanisms such as neuroplasticity remains underexplored. This study aimed to investigate the effects of iTBS using conventional cognitive measures (i.e., MMSE), while also examining the potential role of glymphatic system activity (i.e., DTI-ALPS index). Given that neuroplasticity is relatively preserved in early AD [32], we hypothesized that iTBS would demonstrate immediate and delayed cognitive benefits,, potentially accompanied by changes in glymphatic activity. This dual approach focuses on the cognitive improvements observed from iTBS, providing insight into the longer-term physiological mechanisms, potentially leading to advanced therapeutic strategies for MCI and early AD.

## Methods

### Study design and participants

We conducted a double-blind, randomized, sham-controlled trial to investigate the neuromodulatory effects of iTBS on cognitive function and glymphatic activity in individuals with amnestic MCI and very mild AD. From October 2020 to March 2022, we screened 121 individuals at the Department of Neurology Clinic, Taipei Medical University-Shuang Ho Hospital. Inclusion criteria required participants to be between the ages of 40 and 80 years old with normal or corrected-to-normal vision. They had to meet the core criteria for mild or major neurocognitive disorder due to AD, as outlined in the fifth edition of the Diagnostic and Statistical Manual of Mental Disorders (DSM-5), diagnosed with MCI or very mild dementia due to AD by neurologists based on the criteria proposed by National Institute on Aging and the Alzheimer’s Association (NIA-AA) [33], with a clinical dementia rating (CDR) of 0.5. Exclusion criteria included a personal or family history of generalized tonic-clonic seizures; ongoing treatment for critical conditions; a history of drug or alcohol addiction; being a supervised student or research assistant of the principal or co-principal investigator; major systemic diseases affecting cognitive function (e.g., heart, lung, liver, or kidney failure; poorly controlled diabetes with HbA1C > 8.5%; traumatic brain injury; stroke; or other neurodegenerative conditions); claustrophobia; the presence of metal implants; use of medications that may lower the seizure threshold; fear of TMS; specific allergies; pregnancy or breastfeeding; and a history of suicide attempts or current suicidal ideation. To specifically include amnestic MCI participants, MCI participants presented with a relatively preserved memory function, as assessed by comparing the participant’s neuropsychological measures to age- and education-adjusted normative means, were also excluded. The Geriatric Depression Scale (GDS) was used for depression evaluation [34]. The trial was approved by the Taiwan Food and Drug Administration (TFDA 1096015534) and the Taipei Medical University Joint Review Board (TMU-JIRB No. N202003022) and registered at Clinicaltrials.gov (NCT04555941).

### Sample size Estimation

Sample size estimation was performed using G*Power software version 3.1 [35]. Based on a previous study [36] employing 10 sessions of HF-rTMS at the precuneus in individuals with early AD, an effect size of 0.268 was determined. The study design involved 2 parallel groups with 3 measurements and a hypothetical correlation of 0.7 among repeated measurements. We estimated a sample size of 56, which would provide a statistical power of 80% at an α level of 0.05 for a 2-tailed test. To account for a potential 10% dropout rate, 60 participants were required, with 30 in each group.

### Randomization, concealment, and blinding

After signing the informed consent form, eligible participants were randomly assigned in a 1:1 ratio to either the active or sham iTBS group using Microsoft Excel’s random number generator. A researcher not involved in the assessments or stimulations performed the randomization. A licensed clinical neuropsychologist, blinded to the participants’ group allocation, administered neuropsychological tests. Medications that could affect cognition, including benzodiazepines, acetylcholinesterase inhibitors, and selective serotonin reuptake inhibitors, were prescribed as part of participants’ chronic treatment regimens by physicians who were blinded to group allocation. These medications were not adjusted during the trial period, and their dosages remained stable throughout the study to minimize potential confounding effects. The distribution of these medications was comparable between groups, as shown in Table 1. To maintain the integrity of the double-blind design, participants were instructed not to discuss their treatment with other participants or staff. After the study, they were asked to guess whether they had received real or sham stimulation.

**Table 1:** Baseline characteristics of enrolled participants after randomization

Variables	Experimental (*n* = 30)	Control (*n* = 30)
Age (years), mean (SD)	67.8 (7.9)	67.9 (5.7)
Male Sex, n (%)	11 (36.7%)	12 (40.0%)
Education (years), mean (SD)	10.7 (4.2)	10.8 (4.2)
MMSE score, mean (SD)	25.7 (3.0)	26.0 (3.4)
Mild Cognitive Impairment, n (%)	20 (66.7%)	21 (70.0%)
Fazekas score from T2-weighted image, mean (SD)	1.72 (1.51)	1.09 (1.24)
Depression, n (%)	6 (20.0%)	8 (26.7%)
Geriatric Depression Scale, mean (SD)	2.70 (3.30)	3.70 (4.55)
Use of benzodiazepine, n (%)	8 (26.7%)	10 (33.3%)
Use of acetylcholinesterase inhibitor, n (%)	12 (40.0%)	9 (30.0%)
Use of selective serotonin reuptake inhibitor, n (%)	1 (3.3%)	3 (10.0%)

MMSE, Mini-Mental State Examination

### iTBS interventions

iTBS was conducted using a Magstim Rapid2 transcranial magnetic stimulator (Magstim, Whitland, UK) with a standard 70-mm figure-of-eight coil. Based on previous studies [37], the iTBS protocol involved bursts of 3 pulses at 50 Hz, repeated every 200 ms (i.e., 5 Hz) for 2 s. A 2-second train of iTBS was repeated every 10 s, delivering 600 pulses per run. Three runs were conducted per session, with a 5-minute break between each, totaling 1800 pulses over a 20-minute treatment session [38, 39]. Participants underwent 10 sessions of iTBS over two consecutive weeks (Monday to Friday). The stimulation intensity was set at 80% of the resting motor threshold (RMT). The target location was the left dorsolateral prefrontal cortex (DLPFC), known for its evidenced impaired cortex plasticity [40] and positive response to HF-rTMS in previous studies involving individuals with MCI and AD [9, 19, 41–43]. The Brainsight TMS neuronavigation software (Rogue Research, Montreal, Canada) with individualized MRI images was used to ensure accurate stimulation [44, 45], with a radius of 10 mm around the coordinates [− 38 44 26] based on the Montreal Neurological Institute (MNI). For the sham group, a Magstim placebo coil was used to simulate the sound and sensation of stimulation without delivering an actual magnetic pulse.

### Procedures

**Fig. 1:**
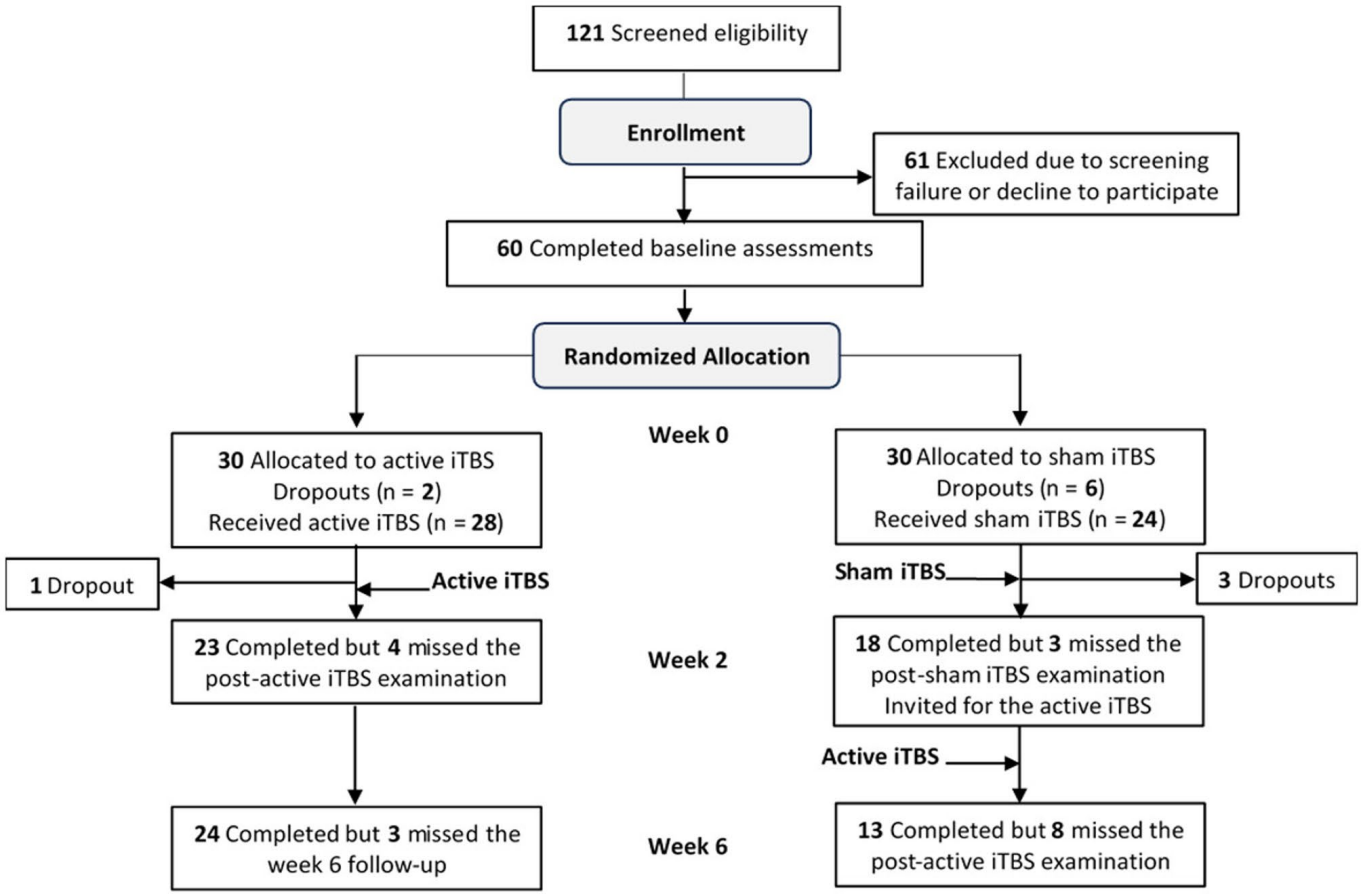
Flowchart of participant enrollment, group allocation, and the timeline for pre-iTBS and post-iTBS evaluations

This randomized controlled trial involved three examinations for each participant: one at baseline, one after the intervention at the end of week 2, and one follow-up at week 6 (Fig. 1). Participants in the experimental group received the active iTBS during the first two weeks, whereas those in the control group underwent sham stimulation during the same period. After the first two weeks, the control group was offered active iTBS treatment in weeks 4 and 5 while the experimental group did not receive further treatment. This sequence ensured that both groups had equal opportunities for real treatment, addressing ethical concerns. A crossover design was not used because previous rTMS studies have shown that participants with experience of rTMS can often distinguish between active and sham stimulation based on sensory perception, making such designs of limited value [46, 47].

### Primary and secondary outcomes

The primary outcome was the MMSE score, while the secondary outcome was the ALPS index derived from the T1-weighted and diffusion-weighted images of brain MRIs. These assessments were made at baseline, post-intervention after week 2, and at week 6. A systematic review and meta-analysis found that the effects of consecutive rTMS sessions can last for at least 4 weeks [48]. Since neuroplasticity-related changes may take time to appear, we assessed the experimental group at week 6 (4 weeks after active iTBS) to explore delayed effects on cognition and glymphatic activity.

### Brain MRI acquisition and image analysis

Brain images were acquired using a 3.0-T GE Discovery MR750 scanner (GE Healthcare, Chicago, IL). The Statistical Parametric Mapping (SPM 12; https://www.fil.ion.ucl.ac.uk/spm/*)* toolbox and ExploreDTI software were used for image preprocessing and diffusion tensor image (DTI) analysis. The diffusion-weighted images (DWI) were coregistered to T1-weighted images. The resulting images were then fitted to the DTI model. Each participant’s fractional anisotropy (FA) map was coregistered to the FA map template in the MNI space. We extracted the periventricular projection and association fibers 25 to 33 mm above the anterior–posterior commissure line in the MNI space [29]. The ALPS-index was then calculated as Taoka et al. described [30]. The entire image preprocessing, visualization, and ALPS index calculation pipeline was executed using MatLab (version R2022b; Natick, Massachusetts, USA). Qualitative assessments of the presence and severity of white matter hyperintensities on T2-weighted images at baseline were performed by either a neurologist or a radiologist using the Fazekas scoring system [49].

### Statistical analyses

Normality tests were conducted prior to all the analyses. Baseline MMSE scores and ALPS indices between the 2 groups were compared using the Mann–Whitney test and independent sample *t* test, respectively. After two weeks, changes in MMSE score (week 2 MMSE – baseline MMSE) and ALPS index changes (week 2 ALPS – baseline ALPS) between groups were also compared using the Mann–Whitney test. Additionally, paired sample *t* tests were used to assess changes in MMSE scores before and after active iTBS intervention, combining data from the experimental group (week 2 – week 0) and the control group (week 6 – week 2). Wilcoxon tests were used to compare ALPS indices before and after active iTBS intervention. Third, MMSE scores and ALPS indices in the experimental group at baseline and week 6 were compared using paired sample *t* tests. All analyses were conducted using MedCalc (version 20.114). All hypotheses were tested at a two-sided significance level of 0.05.

## Results

### Characteristics of the study population

Of the 121 participants screened, 60 were enrolled, completed baseline examinations, and randomized into either the experimental (*n* = 30) or control group (*n* = 30) (Fig. 1). Of these, 28 participants in the experimental group and 24 in the control group had completed the pre-intervention brain MRI. After two consecutive weeks of treatment, 23 participants in the experimental group and 18 in the sham group completed their evaluations. By the end of the study, 24 participants in the experimental group and 13 in the control group completed the 6-week follow-up. Participants were then asked to identify whether they believed they had received active or sham stimulation during the first 2 weeks. A substantial proportion of participants in both the real (88%) and sham (88.5%) groups believed they had received real stimulation, and there was no statistically significant difference between groups (χ2 [1, *N* = 51] = 0.147, *p* =.701), indicating effective blinding.

Table 1 summarized the baseline characteristics, including demographics, MMSE scores, proportion of individuals with MCI, Fazekas scores from T2-weighted images, and GDS scores, and the proportion of participants taking medications potentially affecting cognition. The comparable distribution of these characteristics and medication between groups supports the adequacy of randomization and reduces the likelihood that such medications confounded the observed group differences in cognitive outcomes. The overall mean MMSE score was 25.9 ± 3.2, reflecting relatively high baseline cognitive function among the participants.

### iTBS efficacy

**Fig. 2:**
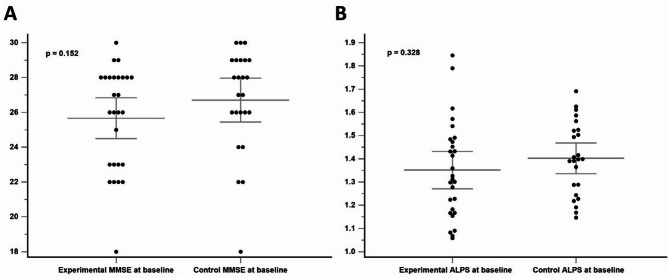
Comparison of baseline MMSE scores (**A**) and ALPS indices (**B**) between the experimental and control groups

**Fig. 3:**
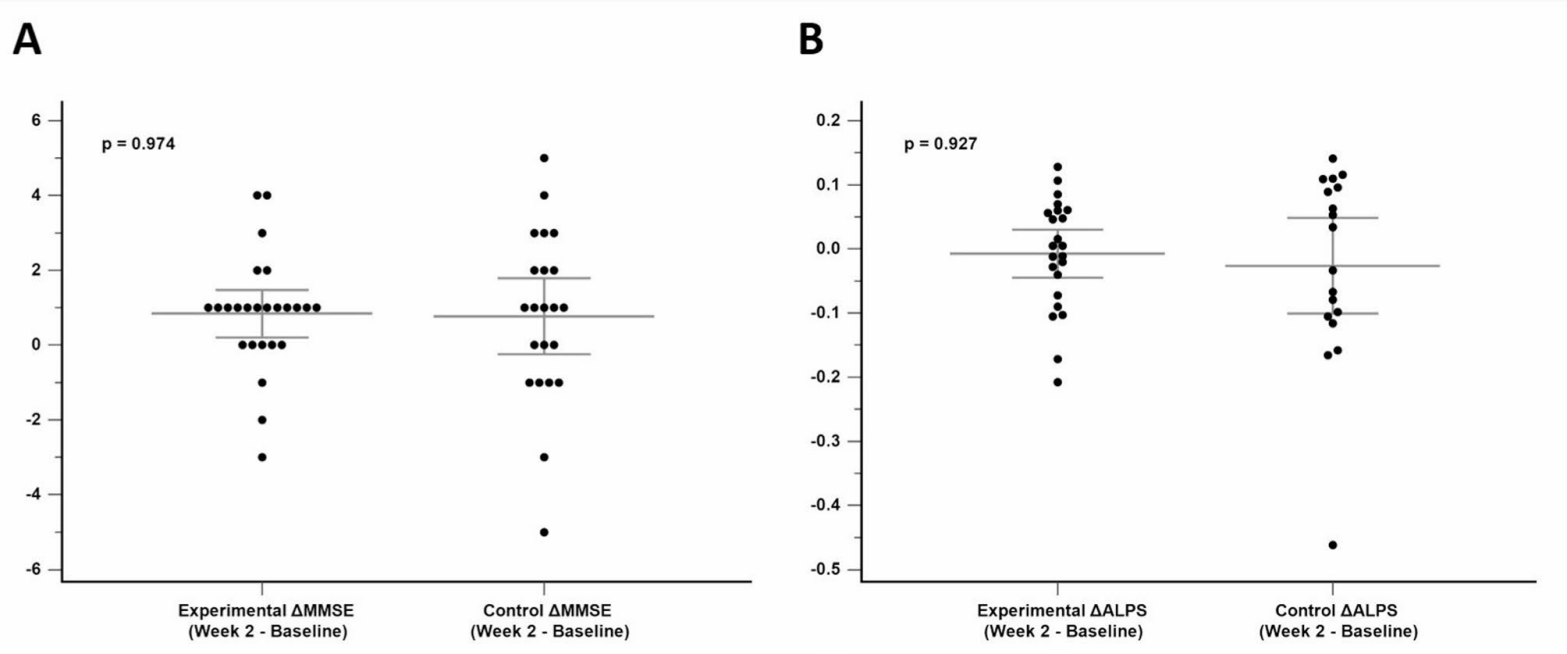
Comparison of MMSE score changes (**A**) and ALPS index changes (**B**) at week 2 between the experimental and control groups

**Fig. 4:**
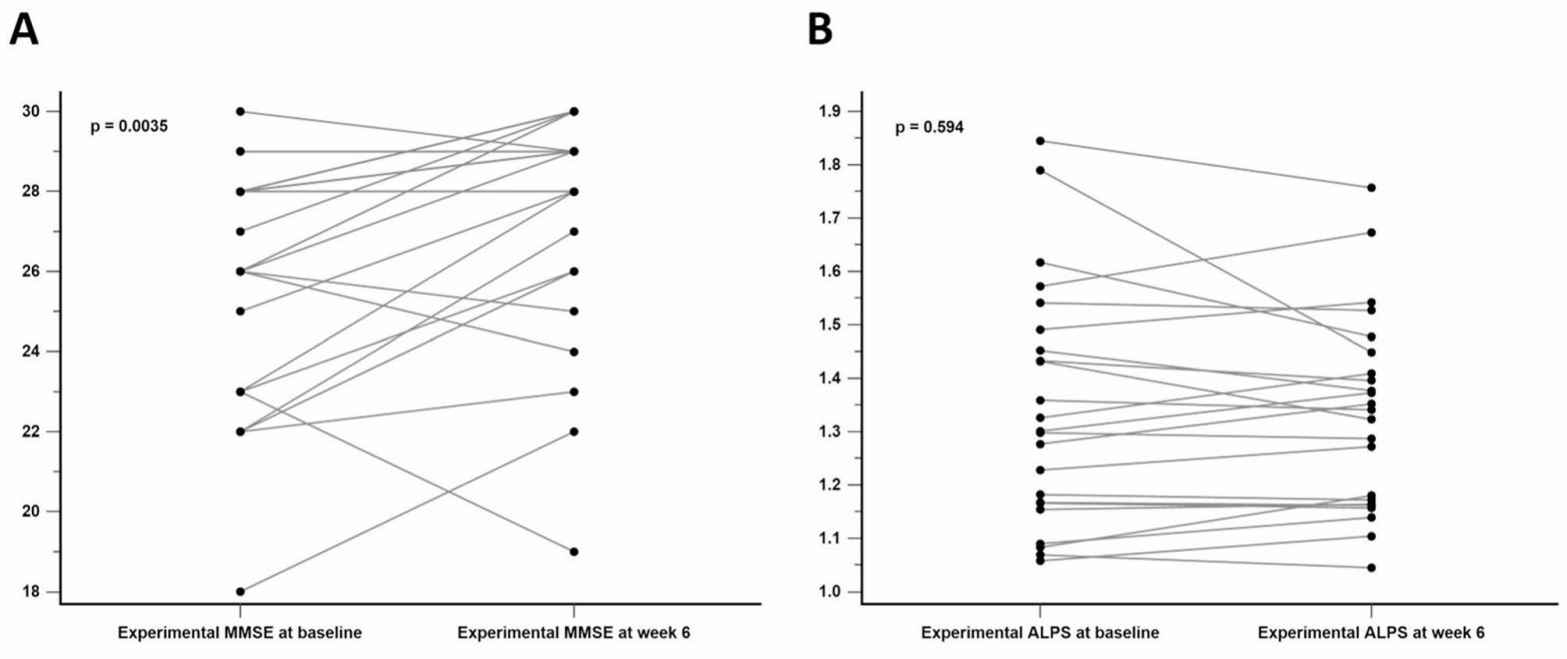
MMSE scores (**A**) and ALPS indices (**B**) before and after 2 weeks of active iTBS in the experimental group

No significant group differences were found in baseline MMSE scores (*P* =.152) or ALPS indices (*P* =.328) for the two groups (Fig. 2). At the end of week 2, neither group showed changes in MMSE scores (*P* =.974) nor changes in ALPS indices (*P* =.927) (Fig. 3). When combining the data from the active stimulation of the experimental group (week 2 - week 0) and the control group (week 6 - week 2), no significant differences were observed in MMSE scores (mean pre-iTBS MMSE score = 26.26; mean post-iTBS MMSE score = 26.63; *P* =.258) or ALPS indices (mean pre-iTBS ALPS value = 1.37; mean post-iTBS ALPS value = 1.35; *P* =.469) before and after 2 weeks of real iTBS treatment (Fig. 4). These results suggest that iTBS did not yield immediate cognitive or glymphatic system benefits in individuals with amnestic MCI or very mild AD.

### iTBS delayed effect

**Fig. 5:**
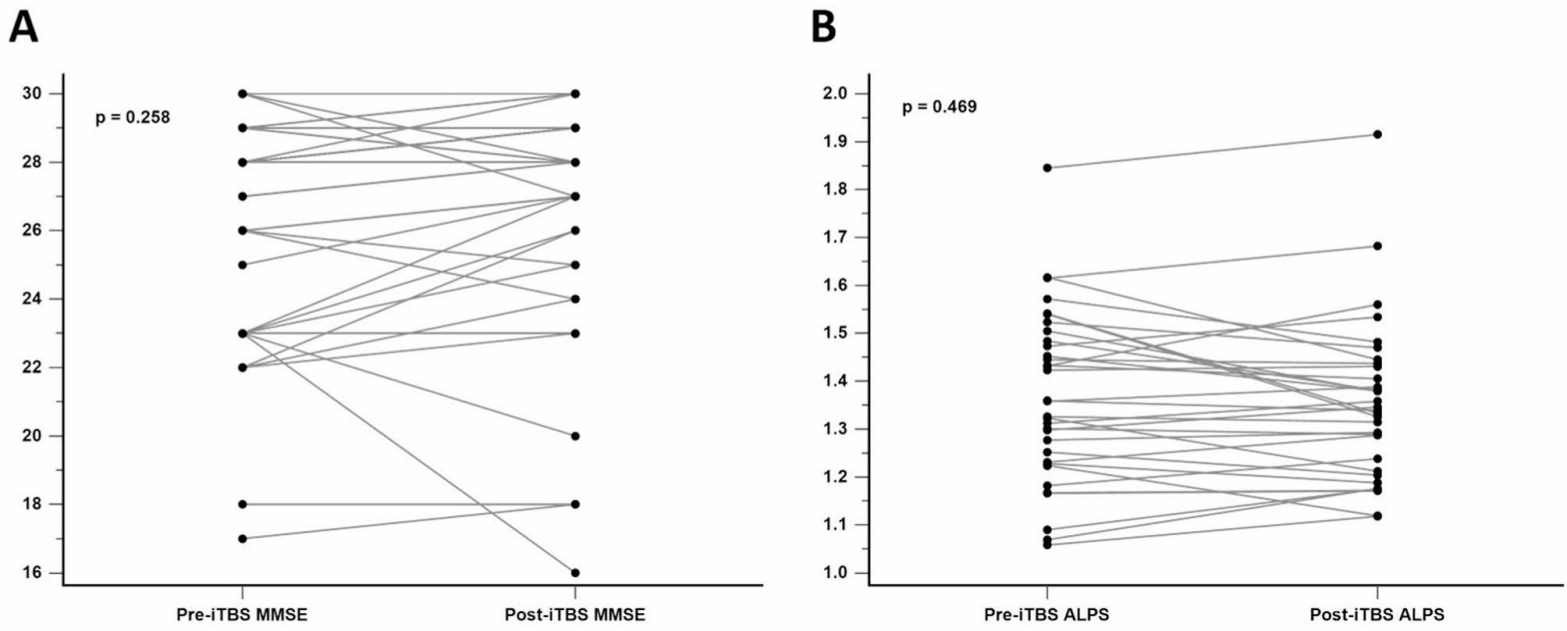
(**A**) Significant increase in MMSE scores at week 6 compared to baseline in the experimental group. (**B**) No significant difference in ALPS indices between week 6 and baseline in the experimental group

At week 6, participants in the experimental group showed a significant improvement in MMSE scores compared to baseline (mean baseline MMSE score = 25.78; mean MMSE score at week 6 = 27.35; *P* =.0035; Fig. 5a). As a comparison, the mean MMSE score change in the control group between baseline and week 6 was − 0.14 (*P* =.751). However, no significant difference in ALPS indices was observed between baseline and week 6 (*P* =.594; Fig. 5b). These results suggest that the iTBS used in this study may produce delayed cognitive improvements but has no impact on glymphatic system activity in individuals with amnestic MCI or very mild AD.

### Adverse events

No adverse events related to iTBS were reported. One participant withdrew due to suicidal ideation after the first sham stimulation session. Other dropouts were attributed to other commitments, COVID-19 restrictions, or withdrawal of consent, none of which were related to iTBS treatment.

## Discussion

This study aimed to examine whether two weeks of iTBS could enhance cognitive function and modulate the glymphatic system in individuals with amnestic MCI and very mild AD. First, compared to the sham group, active iTBS did not significantly improve MMSE scores immediately after the two-week stimulation period. Second, consistent with these cognitive findings, no significant changes were observed in the ALPS index between the experimental and control groups at the end of week 2. However, a noteworthy finding was the significant improvement in MMSE scores at week 6 in the experimental group, four weeks after the active iTBS treatment, compared to baseline. Notably, the control group, which received sham stimulation during weeks 0–2 and only began active iTBS at week 4, did not show cognitive improvement at week 6. Therefore, the delayed cognitive benefit appears specific to the initial active iTBS period and cannot be explained by nonspecific factors such as time effects, testing familiarity, or placebo response. Despite this delayed cognitive enhancement, no significant changes in the ALPS index were found at week 6. This suggests that while the iTBS protocol used in our study may produce delayed cognitive benefits, it does not appear to impact glymphatic system activity, as measured by the ALPS index, in individuals with amnestic MCI and very mild AD.

Studies have shown that the cognitive augmentation effects of HF-rTMS can appear immediately after treatment or take up to a month to manifest [8, 9, 37]. This could be due to the prolonged neuromodulatory impacts of iTBS, as it induces long-lasting changes in synaptic plasticity [50, 51]. The theoretically delayed onset of neuroplastic changes induced by neuromodulation might take time to develop fully [52, 53]. It would explain the cognitive improvements seen at follow-ups rather than immediately after treatment across various clinical populations [52, 54–57]. Another factor contributing to the delayed cognitive improvement may be the relatively high baseline MMSE scores of our participants. With higher initial cognitive function, there may have been a “ceiling effect,” limiting the room for immediate improvement. The delayed effect at week 6, without immediate gains post-iTBS, suggests a true cognitive enhancement rather than a mere practice effect. This highlights the need to assess outcomes at multiple follow-up points to capture the full therapeutic potential of iTBS, recognizing that neuroplastic effects can take time to emerge [53, 55].

Contrary to previous research demonstrating a positive correlation between the ALPS index and cognitive performance [30], our study did not observe significant changes in ALPS indices four weeks post-iTBS in individuals with amnestic MCI and very mild AD. Moreover, no immediate increase in ALPS indices was detected. Several factors may explain our results. First, the ALPS index, which is derived from a specific ROI, may not fully capture regional changes in glymphatic system that could be modulated by iTBS. While iTBS is believed to enhance brain function and induce neuroplasticity through long-term potentiation [13–15, 17], these neuromodulatory changes in glymphatic system activity may occur in regions outside the ROI from which the ALPS index is derived. Second, while HF-rTMS has improved glymphatic drainage in animal models of AD [31], it is continuous TBS (cTBS) rather than iTBS that has demonstrated positive effects on the glymphatic system in animal studies [58]. It is hypothesized that cTBS modulates glymphatic activity through activation of the inhibitory γ-aminobutyric acid (GABA) system [59]. However, since iTBS has shown positive effects in both animal [60] and human studies [37], its benefits in AD-related pathology may be less associated with the glymphatic system. Third, the variability of the ALPS index in cognitively preserved individuals may limit its sensitivity to change in glymphatic system activity in our study cohort, which had relatively high MMSE scores [30]. Fourth, recent studies suggest that the ALPS index may primarily reflect Brownian motion of water molecules in the radial direction at the level of the lateral ventricular body, which may only partially capture glymphatic activity [61]. To provide a more comprehensive assessment of glymphatic activity, future studies may consider combining the ALPS index with other imaging methods such as free water analysis, perivascular space volume quantification, or functional MRI.

The selection of the left DLPFC as the stimulation target was well-supported by previous studies. The DLPFC plays a pivotal role in executive control and is part of critical frontoparietal networks that underlie cognitive processes [62–64]. This region is also vulnerable to AD-related changes, making it an ideal target for interventions aimed at enhancing synaptic plasticity and improving cognitive function [14, 40, 65]. Studies have shown that iTBS targeting the left DLPFC is effective and safe, with minimal adverse effects [66]. Therefore, selecting the left DLPFC as the target is both logical and supported by its established role in cognitive enhancement [67, 68].

Despite the promising results, several limitations of our study should be noted. First, although our sample size was reasonable, larger multicenter studies are necessary to validate our findings. Second, the COVID-19 pandemic caused interruptions, leading to dropouts and missed follow-up appointments. However, our dropout rates were comparable to those of other iTBS studies conducted under challenging conditions [37]. Third, while advances in biomarkers for AD diagnosis have been significant [69], our reliance on symptomatic clinical approaches may limit the internal validity of the study [70]. Future research should consider including more advanced biomarkers to provide a clearer picture of iTBS effects on glymphatic activity. Lastly, the iTBS parameters used in this study were based on prior validated protocols [37–39], ensuring feasibility and tolerability in an outpatient setting. While our findings support the clinical potential of this protocol in producing delayed cognitive benefits in individuals with amnestic MCI and very mild AD, the optimal stimulation configuration for this population has yet to be established. Adjustments in stimulation intensity, session number, or inter-session intervals may further enhance or accelerate cognitive outcomes, reduce inter-individual variability, and potentially affect other domains such as glymphatic function. The FDA-approved Stanford Accelerated Intelligent Neuromodulation Therapy (SAINT) applies ten iTBS sessions per day with 50-minute inter-session intervals to engage metaplastic mechanisms. However, this highly intensive schedule may limit applicability in older adults with MCI or very mild AD, particularly in outpatient settings. In contrast, the 5-minute interval used in our protocol was derived from previous clinical studies in depression populations [38] and was selected to ensure tolerability. Existing literature suggests that the effects of repeated iTBS with varying inter-session intervals may differ across individuals, with some studies reporting facilitation while others observe diminished or reversed responses [71, 72]. Although our study did not directly compare stimulation intervals, the delayed cognitive improvement observed at week 6 suggests that even shorter-duration interventions may elicit meaningful neuromodulatory effects over time. Future research should directly examine how different iTBS spacing intervals influence cognitive and physiological responses to determine the most effective and practical approach for individuals with MCI or early AD.

## Conclusions

In conclusion, the iTBS protocol used in our study demonstrated delayed cognitive enhancement in individuals with amnestic MCI or very mild AD, supporting its potential clinical applicability in this population. However, the protocol did not appear to modulate glymphatic system activity as measured by the ALPS index. These findings suggest that iTBS may offer cognitive benefits through mechanisms independent of glymphatic function, or that current imaging approaches may lack sensitivity to detect such changes. Future randomized trials with larger samples and refined protocols with variations in session spacing, stimulation intensity, or total dose are warranted to optimize therapeutic outcomes and further elucidate the mechanisms of action, potentially through multimodal imaging and biomarker-based assessments.

## Data Availability

The data are available from the authors upon reasonable request and with the permission of the corresponding author.

## Abbreviations

AD: Alzheimer’s Disease
ALPS: Analysis along the Perivascular Space
CDR: Clinical Dementia Rating
COVID-19: Coronavirus Disease 2019
cTBS: Continuous Theta-Burst Stimulation
DLPFC: Dorsolateral Prefrontal Cortex
DSM-5: Diagnostic and Statistical Manual of Mental Disorders, Fifth Edition
DTI: Diffusion Tensor Imaging
DTI-ALPS: Diffusion Tensor Image Analysis along the Perivascular Space
DWI: diffusion-weighted images
FA: fractional anisotropy
GABA: Gamma-Aminobutyric Acid
GDS: Geriatric Depression Scale
HF-rTMS: High-Frequency Repetitive Transcranial Magnetic Stimulation
iTBS: Intermittent Theta-Burst Stimulation
MCI: Mild Cognitive Impairment
MMSE: Mini-Mental State Examination
MNI: Montreal Neurological Institute
MoCA: Montreal Cognitive Assessment
MRI: Magnetic Resonance Imaging
NIA-AA: National Institute on Aging and the Alzheimer’s Association
RMT: Resting Motor Threshold
ROI: Region of Interest
rTMS: Repetitive Transcranial Magnetic Stimulation
SAINT: Stanford Accelerated Intelligent Neuromodulation Therapy
TMS: Transcranial Magnetic Stimulation
